# El Niño southern oscillation, weather patterns, and bacillary dysentery in the Yangtze River Basin, China

**DOI:** 10.1186/s41256-024-00389-4

**Published:** 2024-11-11

**Authors:** Caiji Li, Xiaowen Wang, Zehua Liu, Liangliang Cheng, Cunrui Huang, Jing Wang

**Affiliations:** 1 School of Public Health, Sun Yat-Sen University, Guangzhou, 510080 China; 2 Department of Nutrition, Harvard TH Chan School of Public Health, Boston, MA 02115 USA; 3 Vanke School of Public Health, Tsinghua University, Haidian District, 100084 Beijing China

**Keywords:** El Niño southern oscillation, Bacillary dysentery, Climate variability, Weather pattern, Mediation analysis

## Abstract

**Background:**

Increasingly intense weather anomalies associated with interannual climate variability patterns, like El Niño-southern oscillation (ENSO), could exacerbate the occurrence and transmission of infectious diseases. However, research in China remains limited in understanding the impacts and intermediate weather changes of ENSO on bacillary dysentery (BD). This study aimed to reveal the relationship between ENSO, weather conditions, and the incidence of BD, and to identify the potential meteorological pathways moderated by ENSO in the ENSO-BD connections.

**Methods:**

BD disease data and meteorological data, as well as ENSO index, from 2005 to 2020 were obtained for 95 cities in the Yangtze River Basin. We first established the associations between ENSO events and BD, ENSO and weather, as well as weather and BDs using two-stage statistical models. Then, we applied a causal mediation analysis to identify the specific meteorological changes in the ENSO-BD relationship.

**Results:**

In the Yangtze River Basin, both El Niño (IRR: 1.06, 95%CI: 1.04 ~ 1.08) and La Niña (IRR: 1.03, 95%CI: 1.02 ~ 1.05) events were found to increase the risk of BD. Variations of ENSO index were associated with changes in local weather conditions. Both the increases in regional temperatures and rainfall were associated with a higher risk of BD. In the casual mediation analyses, we identified that higher temperatures and excessive rainfall associated with La Niña and El Niño events mediated the ENSO’s effect on BD, with mediation proportions of 38.58% and 34.97%, respectively.

**Conclusions:**

Long-term climate variability, like ENSO, can affect regional weather conditions and lead to an increased risk of BD. We identified the mediating weather patterns in the relationship between ENSO and BD, which could improve targeted health interventions and establish an advanced early warning system in response to the BD epidemic.

**Supplementary Information:**

The online version contains supplementary material available at 10.1186/s41256-024-00389-4.

## Background

The increasing trends in rising temperatures and intensified hydrological events due to climate change pose great threat to human health [1]. In particular, these changes in climate variability and shifts in weather conditions at different time scales can alter the natural environments, ecosystems, and social vulnerability, which could affect the probability of a range of disease outbreaks [2]. With its climate diversity and huge population, China is espeicially vulnerable to climate change and is projected to suffer more adverse health impacts, thereby undermining its progress in reducing the burden of infectious diseases [3]. Yet, despite intensive research into relationship between infectious diseases and certain meteorological factors, it is still challenging to study the impacts of climate variability from a long-term perspective [4].

As a major driver of global interannual climate variability, the El Niño-southern oscillation (ENSO) phenomenon is characterized by periodic fluctuations of sea surface temperatures (SSTs) and atmospheric conditions in the central equatorial Pacific Ocean. The periodic swing in SSTs anomalies between positive to negative extremes leads to two anomalous conditions known as El Niño (warm) and La Niña (cold) events, and the cycle often lasts over 2–7 years. The circulation of SSTs and ENSO events is often accompanied by extremes in local weather patterns such as temperatures, monsoons, and precipitation across the globe [5]. Although ENSO is originated from the central Pacific Ocean, it has far-reaching consequences around the world. ENSO has profound impacts on global climate and weather anomaly patterns, often defining major peaks in spatial and temporal dimensions of extreme weather conditions, especially for those places that are tele-connected to ENSO like South America, East Asia and Africa [6]. Because of the dominant position of the global climate phenomenon affecting meteorological conditions, ENSO is viewed as a natural experiment to observe the impacts of large-scale climate variability on myriad subjects, such as agricultural production [7], economic output [8], social stability [9], and human health [10].

The adverse impacts of ENSO on human health have been widely studied, among which the infectious diseases are paticularly important because nearly two-thirds of them are climate-sensitive [11]. The regional surges in weather anomalies following ENSO events can affect the pathogens, route of transmission, and hosts through various ways, which could drive the occurrence and exacerbate the spread of infectious diseases [12]. Moreover, given that ENSO conditions can be predicted months in advance through climate forecasting [13], it would be meaningful to directly link ENSO with a range of infectious diseases. Therefore, it is necessary to develop an improved understanding of the impacts of ENSO on infectious diseases for optimized preparedness in disease forecast and prevention.

Although several studies have linked ENSO to infectious diseases including dengue fever [14], malaria [15], and diarrhea [16], most have focused on a single region or one ENSO phase, resulting in inconsistent findings. In Bangladesh [16], they found positive associations between cholera and El Niño-related regional temperature anomalies, while studies in Nepal [17] and Botswana [18] found that La Niña-related weather anomalies may drive an increase in local rainfall, thus promoting the incidence of diarrhea in children under five. These findings indicated a complex relationship between ENSO variability and disease transmission, as the associations will be mediated by local weather pathways that are spatially distinct in different ENSO teleconnected places. In China, evidence of the effect of ENSO on noteworthy infectious diseases such as infectious diarrhea still remain limited [19], with only one empirical study exploring ENSO-diarrhea relationship in Shandong province, which failed to demonstrate the local weather changes moderated by ENSO [20]. In exploring the impacts of ENSO on infectious diseases, identifying such potential intermediate weather patterns would help the public services to implement more practical and targeted measures for disease interventions.

To address the knowledge gap in current research, we investigated how ENSO-moderated weather changes are associated with bacillary dysentery. Bacillary dysentery (BD) is a type of acute infectious diarrhea, which to date remains a serious public health burden in China, ranking as the third most reported infectious disease [21]. As one of the climate-sensitive infectious diseases, BD is mainly transmitted through food, water, and person-to-person contact, which are highly affected by meteorological factors [22]. Since the evidence of climate teleconnection with ENSO was well-documented, we conducted a regional study in the Yangtze River Basin, China, where ENSO variability often resulted in changes in terms of temperature and rainfall anomalies [23]. In addition, the Yangtze River Basin has the highest prevalence of BD, accounting for nearly one-third of the annual reported cases in the country [24]. In this study, we aimed to establish the associations between ENSO-BD, ENSO and weather, and weather conditions and BD through a long-term dataset. We also aimed to identify the intermediate weather pathways in the ENSO- BD associations through causal mediation analysis, to improve the ability to predict and control diarrhea risk under future climate change.

## Methods

### Study area

**Fig. 1:**
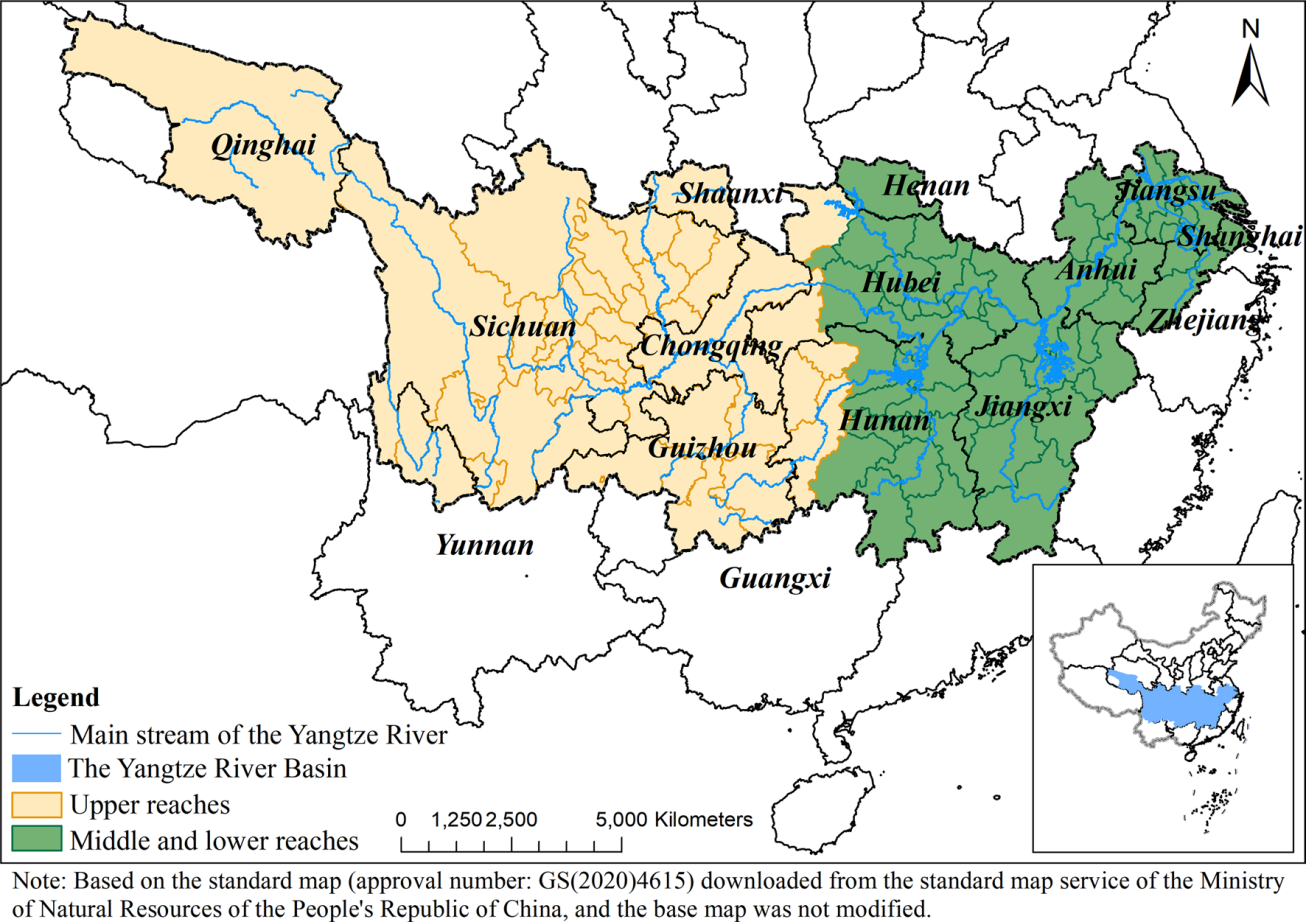
Map of study area in the Yangtze River Basin, China

The Yangtze River Basin is one of the most populated regions in China, home to more than one-third of the nation’s population [25]. With a multi-tier terrain and influenced by the summer monsoon, the Yangtze River Basin has complex climate conditions, as most of the basin is located in subtropical and temperate climate zones, resulting in a hot and humid summer and a cold and dry winter [23]. Based on its varied geomorphology, the Yangtze River Basin is usually divided into the Upper, Middle and Lower reaches (separated by the city of Yichang). The Upper reaches of the Yangtze River Basin are located in a cold and dry high-altitude area, governed by a semi-arid and semi-humid climate, while the Middle and Lower reaches are dominated by plains with warm and humid climates. There exited significant differences in social conditions between the Upper reaches and the Middle and Lower reaches [26]. The risk of the disease depends not simply on meteorological conditions, but also the underlying social and ecological contexts [21]. As a result, we divided the area into Upper, Middle and Lower reaches based on geographic differences in order to further control the social and ecological factors. Under the guideline of spatial statistic trinity [27], we additionally quantified the spatial characteristics of BD in those different reaches. We selected a total of 95 prefecture-level cities (including 2 municipalities) within the Yangtze River Basin in this study, with 39 located in the Upper reaches and 56 in the Middle and Lower reaches (Fig. 1).

### Data collection

The time-series monthly Niño 3.4 index data were obtained from the National Oceanic and Atmospheric Administration of the United States (http://www.cpc.ncep.noaa.gov/), representing a three-month running mean of sea surface temperatures in the equatorial Pacific (5°N–5°S, 170–120°W) [28]. According to the *Identification method for El Niño/ La Niña events* issued by the China Meteorological Administration [29], ENSO events were classified based on Niño 3.4 SST anomalies, for El Niño events (warm events) were defined as Niño 3.4 index above 0.5 K for five consecutive months, and La Niña events (cold events) were defined as Niño 3.4 index below -0.5 K for five consecutive months. We obtained and verified county-level BD individual case data, including age, diagnostic date, and the administrative code of the corresponding county from the China National Notifiable Disease Surveillance System (NDSS). BD is one of the legally mandated category B notifiable diseases. According to the China's national communicable disease control act, all health and sanitation facilities are required to report BD cases through the NDSS. The NDSS has been effectively established and in use since January 2004 [30]. The diagnostic and classification criteria of BD was according to the Chinese diagnostic criteria for infectious diarrhea, the management standards and principles of dysentery issued by the Ministry of Health of the People’s Republic China [31]. We then aggregated the data into monthly time-series BD cases by city for the following analyses. The annual city’s average population was obtained from the China Statistical Yearbook (http://www.stats.gov.cn/tjsj/ndsj) during study period to allow for comparability. The meteorological data including daily maximum temperature, relative humidity, and precipitation during the study period were obtained from the fifth generation of the European Re-Analysis (ERA5) dataset [32]. We extracted the 0.25*0.25 degrees resolution of grid weather data and matched them with the corresponding cities by longitude and latitude. Then, we aggregated them to monthly time-series data from January 2005 to December 2020.

## Data analysis

Under the guidelines of the SST, first, we measured the spatial stratified heterogeneity of BD in the Yangtze River Basin by applying a Geo-Detector method. Geodetector is an advance statistical method to detect spatial stratified heterogeneity and reveal the driving factors behind it [33]. *Q*-statistic in Geo-detector has already been applied in many fields of natural and social sciences to measure spatial stratified heterogeneity [34]. The *q*-statistic is a monotonic function of the strength of the spatial stratified heterogeneity [35]. The result is listed in Additional File 1, Table S1. Geographically, we observed an apparent spatial stratified heterogeneity of BD cases in the Yangtze River Basin, with *q*-statistic value of 0.24. The results indicated that both rainfall and temperature played a dominant role in the spatial heterogeneity of the disease.

### Association analysis

We used a two-stage statistical model for the association analysis to quantify the city-level relationship, and then the pooled associations were obtained at regional and sub-regional levels by meta-analysis. The association model formulations are described below.

Firstly, to estimate the association between the ENSO-BD relationship in the Yangtze River Basin, we used a generalized linear model with quasi-Poisson distribution following previous research [36],

1 $$\log E\left[ {Y_{it} } \right] \sim \beta_{0} + b_{i} + \beta_{1} ENSO_{it} + \beta_{2} TEM_{it} + \beta_{3} RHU_{it} + \beta_{4} PRE_{it} + \begin{array}{*{20}c} {\beta_{5} {\text{Season}}_{it} + ns\left( {{\text{time}}_{it} |df} \right) + \log POP_{it} ,} \\ \end{array}$$

where ${Y}_{it}$ stands for monthly counts of BD cases in city *i* during month *t*. The ${ENSO}_{it}$ refers to categorical variable indicating different ENSO phases (0 for ENSO-normal phase as reference) occurred in city *i* during month *t.* The weather conditions were controlled in linear terms for *TEM* as monthly maximum temperature, *PRE* as monthly precipitation, and *RHU* as monthly mean relative humidity in the corresponding month and city in the model. *Season* was a four-level categorical variable (Winter, Spring, Summer, Fall) categorized by different seasons (December – February, March – May, June – August, September—November). As for the long-term trend analysis, we applied a natural cubic spline for month numbers, with 16 degrees of freedom accounting for 16 years of study period based on previous research [36]. We also used an offset ${POP}_{it}$ in the model to control the differences in each city’s population. The ${b}_{i}$ was used as a random intercept to account for city baseline risks. The cumulative 0–3 lag effects were estimated separately for each ENSO event and reported as incidence rate ratios (IRR) with 95% confidence intervals (95%CI).

Secondly, we used grid-level monthly total precipitation data and the Niño 3.4 index to identify the teleconnection between ENSO and local weather [37]. Correlations were estimated within each grid cell for different seasons. The area in the Yangtze River Basin was tele-connected to ENSO when the gridded precipitation in month t is significantly correlated with Niño 3.4 index of the season at the second month lag (lag t-2 month).Then, We assessed seasonal associations between Niño 3.4 index and regional monthly rainfall, monthly maximum temperature based on linear regression with gaussian distribution (Model 2). Following the previous literature, we used Niño 3.4 index to predict the seasonal changes of rainfall and temperature, as well as the lagged effect of ENSO index [38]. The results were calculated by separate models stratified by four seasons, using lagged 0–12 months Niño 3.4 index

2 $$\begin{array}{*{20}c} {E\left[ {Y_{it} } \right]\sim \alpha + \beta_{1} Ni\ n o_{it} + \beta_{2} Season + \in } \\ \end{array}$$

Thirdly, we applied a generalized linear model with quasi-Poisson distribution at monthly timescale to establish the associations between rainfall, temperature and BD. We calculated a long-term monthly average data for each calendar month specific to each city based on a 30-year period (1991–2020). Then, we calculated the monthly weather anomalies as deviations in given city from the long-term average data for corresponding month of the year. The weather events were then categorized into five groups based on the given anomalies, which above or below the corresponding percentile of each city throughout the study period following previous researches [17]. Event types were specified as 5 categories: a) the normal weather event was a month when the anomalies between the 33rd and 67th percentile and was used as reference in the model; b) an abnormally weather event was a month when the anomalies was higher (or lower) than 90th (10th) percentile; and c) the cold/warm (dry/wet) event was a month when the anomalies was between 10th to 33rd and 67th to 90th percentile. Then, we modeled

3 $$\log E\left[ {Y_{it} } \right] \sim \beta_{0} + b_{i} + \beta_{1} Anomaly_{it} + \beta_{2} weather_{it} + \beta_{3} season_{it} + \begin{array}{*{20}c} {ns\left( {time_{it} |df} \right) + \log POP_{it} ,} \\ \end{array}$$

where all the variable parameters were defined the same as Model 1 before. While ${Anomaly}_{it}$ was the categorical variable refers to different weather events (0 for the normal event as reference), which categorized different weather anomaly in city *i* at month *t*. The model was adjusted by ${weather}_{it}$, representing either temperature or rainfall based on the corresponding weather event.

We further explored the exposure–response relationship between temperature, rainfall, and incidence of BD at daily timescale by using a generalized linear model combined with a distributed lag nonlinear model (DLNM) [39]. We modeled

4 $$\log E\left[ {Y_{it} } \right] \sim \alpha + cb\left( {tem_{it} } \right) + cb\left( {pre_{it} } \right) + ns\left( {rhu_{it} ,3} \right) + ns\left( {{\text{time}}, 7 {\text{per year}}} \right)\begin{array}{*{20}c} { + res_{t - 1} ,} \\ \end{array}$$

where ${Y}_{it}$ refers to the daily counts of BD cases on day *t* in city *i*. Daily maximum temperature was included using a cross-basis natural cubic spline function (*df* = 3) for both the response and lag dimensions. Daily total precipitation was also applied using the same cross-basis natural cubic spline function for both the response and lag dimensions. A lag period of 0–28 days was chosen to capture the delayed effect of weather variables on BD with an average lag period of 1 month [40]. Daily relative humidity ${rhu}_{it}$ was included in the model as a potential confounder. We modeled both the exposure–response and lag-response relationship using natural cubic splines with equally spaced knots and three degrees of freedom chosen from lowest value of the minimum quasi-AIC (akaike information criterion) [41]. A natural cubic spline $ns()$ with 7 degree of freedom per year for time was accounted for seasonality and long-term trends [42]. We additionally included a first-order lagged residual term ${res}_{t-1}$ to control for auto-correlation.

### Causal mediation analysis

Based on the previous association analyses, we conducted a mediation analysis of the ENSO-BD relationship mediated by weather conditions to explore the potential effect modification. Based on the mediation method proposed by Preacher and Hayes [43], we used the regression-based method for binary independent variables to estimate ENSO-BD relationship mediated by temperature and rainfall [44]. The model-based causal mediation analysis was conducted in two steps. First, we modeled generalized linear regressions to estimate the total effect, the average causal mediation effects (ACME), and the average direct effects (ADE) separately for each meteorological mediator. The mediation proportion can be calculated as the ratio of ACME and ADE. Then, the ratio of these estimates interpreted as proportion mediated was calculated to quantify the magnitude of mediation effect for each weather mediator. The mediation analysis was conducted using a bootstrapping approach (1000 samples) [45]. We performed our mediation analysis by using “mediation” package in R software [46].

### Sensitivity analysis

In our study, the following sensitivity analyses were applied to validate the chosen parameters: (1) changing the maximum number of lag months (0, 1, 2, 3) for the effect of ENSO on BD, (2) changing the degrees of freedom (14–16) for the time effect, and (3) changing the df (3–5) for each meteorological factors in the DLNM model. In order to test the stability of ENSO index in the study, we also performed sensitivity analyses by replacing ENSO indices Niño 3.4 into Niño 3 and Niño 4. Results of these analyses are shown in the Additional file Tables S2 and Figure S9- S10.

All the statistical analyses were performed in ArcGIS 10.2 and R software 4.2.1 (R Foundation for Statistical Computing), with packages ‘splines’ (version 4.1) [47], ‘tsModel’ (version 0.4) [48], ‘dlnm’ (version 2.4.6) [49], ‘mediation’ (version 4.5.0) [46] and ‘mvmeta’ (version 1.0.3) [50]. The statistical tests were two-sided, and effects of *P* < 0.05 were considered statistically significant.

## Results

### Descriptive analysis

During the study period, a total of 1,037,150 BD cases were reported from 95 cities in the Yangtze River Basin, accounting for 30.2% of all reported BD cases in the country. Table 1 describes the statistics of the reported BD cases. There were more BD cases in the Middle and lower reaches (547,753; 52.8%) than in the Upper reaches (489,397; 47.2%), and the highest prevalence age group was adults aged 18 to 59-year-old (455,075; 43.8%). In terms of seasonality, 36.7% (381,426) of BD were reported in summer and 28.7% (297,326) were reported in fall. The incidence of bacillary dysentery is higher in the western cities in the upper reaches than cities in the middle and lower reaches (Figure S1b). There was a similar pattern of BD and temperature as well as rainfall, with the cases peaking in summer when the highest temperature and rainfall were recorded throughout the year (Figure S1). On average, monthly rainfall was higher in the entire Yangtze River Basin during the El Niño phase compared to normal and La Niña phase (Table 2). We also observed that the average monthly maximum temperature with La Niña phase is higher than El Niño and normal phase in spring and summer, though remaining lower than El Niño phase in the winter.

**Table 1:** Distribution of BD cases in Yangtze River basin during 2005–2020

Characteristic	Yangtze River basin (95 cities)	Upper reaches (39 cities)	Middle and lower reaches (56 cities)
Total Cases (n, %)	1,037,150	489,397	547,753
Age			
≤ 5	364,843(35.2)	179,979(36.8)	184,864(33.8)
6–17	116,365(11.2)	58,120 (11.9)	58,245(10.6)
18–59	455,075(43.9)	206,337(42.1)	248,738(45.4)
≥ 60	100,867(9.7)	44,961(9.2)	55,906(10.2)
Season			
Spring	226,156(21.8)	116,208(23.8)	109,948(20.1)
Summer	381,426(36.7)	177,489(36.3)	203,937(37.2)
Fall	297,327(28.7)	130,497(26.6)	166,830(30.5)
Winter	132,241(12.8)	65,203(13.3)	67,038(12.2)
ENSO phase			
Normal	540,547(52.1)	253,349(51.8)	287,198(52.4)
El Niño	137,704(13.3)	68,352(14.0)	69,352(12.7)
La Niña	358,899(34.6)	167,696(34.2)	191,203(34.9)

**Table 2:** Meteorological conditions in Yangtze River basin during 2005–2020, stratified by different ENSO phases and season

Monthly weather mean (SD)	Year round	Spring	Summer	Fall	Winter
T_max_ (℃)					
Normal	20.1 (8.4)	21.0 (5.1)	29.1 (4.0)	21.5 (5.5)	9.6 (3.4)
El Niño	19.6 (8.5)	20.2 (4.8)	29.2 (4.1)	19.2 (5.8)	9.9 (3.3)
La Niña	20.4 (8.2)	21.4 (5.2)	29.9 (4.2)	21.3 (5.2)	9.3 (3.7)
Rainfall (mm)					
Normal	117.1 (97.8)	134.6 (90.9)	198.4 (107.1)	88.0 (64.2)	48.3 (45.3)
El Niño	129.5 (104.8)	135.3 (94.6)	208.8 (115.2)	102.9 (62.4)	59.8 (56.9)
La Niña	100.2 (80.4)	112.8 (75.4)	182.7 (90.4)	74.4 (49.6)	43.6 (38.3)

*SD* standard deviation, *T*_max_ maximum temperature.

### Effect of ENSO events on bacillary dysentery

**Fig. 2:**
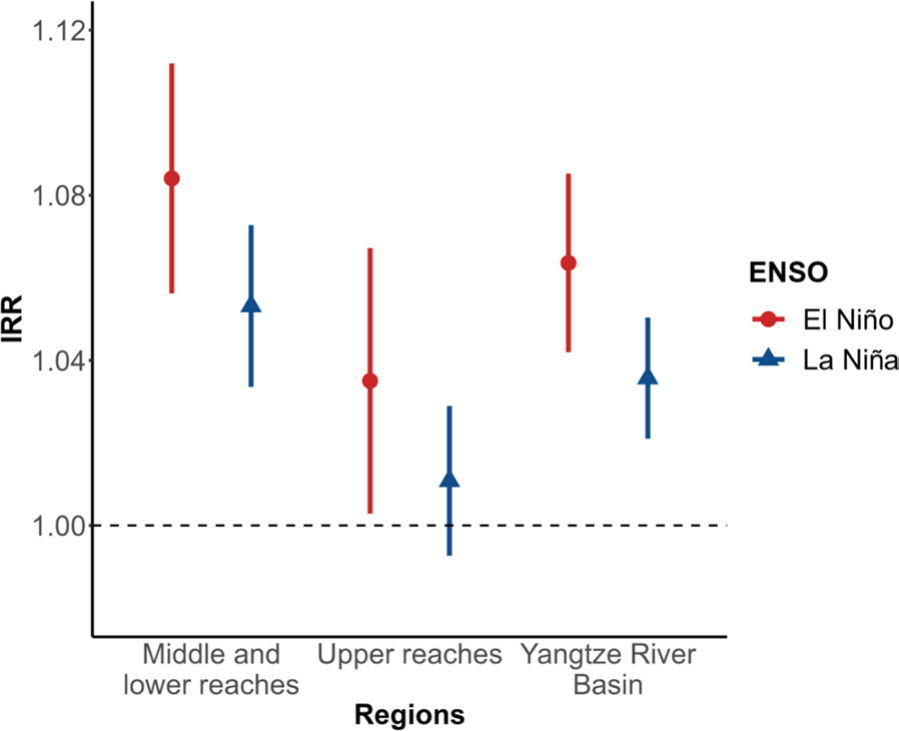
The incidence rate ratios (IRR) and 95% confidence intervals (CI) for the association between ENSO events and BD stratified by different regions

Figure 2 presents the estimated incidence rate ratios of different ENSO events on BD in the Yangtze River Basin. Overall, ENSO events were associated with more BD cases. Compared with normal months, both El Niño (IRR: 1.06, 95%CI: 1.04 ~ 1.09) and La Niña events (IRR: 1.03, 95%CI: 1.02 ~ 1.05) were significantly associated with BD incidence in the Yangtze River Basin. Region-specific results showed that the Middle and lower reaches of the Yangtze River Basin were likely to suffer more negative influence from El Niño events, with a higher incidence rate ratio (IRR: 1.08, 95%CI: 1.06 ~ 1.11) than in the Upper reaches (IRR: 1.03, 95%CI: 1.00 ~ 1.06). We found no significant association between La Niña event and BD incidence in the Upper reaches (IRR: 1.01, 95%CI: 0.99 ~ 1.03), in contrast with the Middle and lower reaches (IRR: 1.05, 95%CI: 1.03 ~ 1.07).

### Association between Niño 3.4 index and temperature, rainfall

**Fig. 3:**
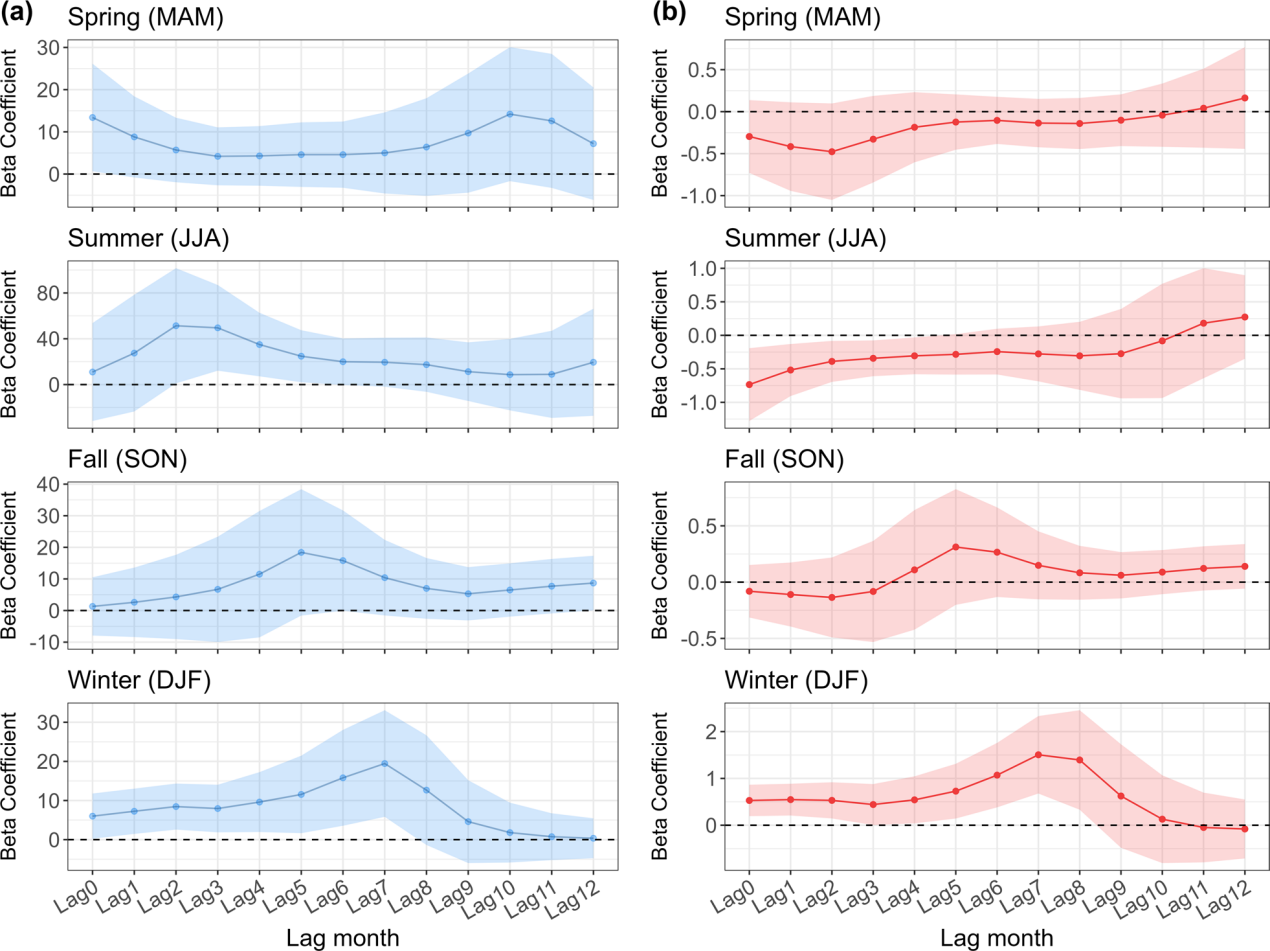
Associations between Niño 3.4 SST anomalies and **a** regional rainfall (mm), **b** maximum temperature (°C) in the Yangtze River basin by different season from 2005 to 2020. Note: The *y*-axis represents Beta coefficients that when change in 1 K (unit) in Niño 3.4 SST anomalies, the corresponding changes in the outcome (in millimeters for rainfall, degrees of Celsius for temperature)

ENSO variability can significantly affect regional climate conditions across different seasons in the Yangtze River Basin. We found a significant positive correlation between the Niño 3.4 index and monthly total rainfall in the majority area of the Middle and lower reaches of the Yangtze River Basin during summer and winter (Figure S2). Further seasonal regression analysis unveiled significant associations among rainfall, temperature, and the Niño 3.4 index across different seasons at different time lags. In the entire Yangtze River Basin, Niño 3.4 index had a positive association with rainfall throughout the year and was significantly associated with rainfall in summer and winter at a 2–5 months lag and 0–7 months lag, respectively (Fig. 3a). The results indicated that the El Niño-like (warmer) Niño 3.4 index was associated with higher regional rainfall. During summer, a 1 K increase of Niño 3.4 index was significantly associated with a 49.5 mm (95%CI: 12.2 ~ 86.9 mm) increase in rainfall at 2-month lag. As in winter, a 1 K increase of Niño 3.4 index led to a 19.8 mm (95%CI: 6.0 ~ 33.5 mm) increase in rainfall at a 7-month lag. Likewise, we found that Niño 3.4 index was significantly associated with average maximum temperature during summer and winter at 0–4 months lag and 0–8 months lag, respectively (Fig. 3b). Specifically, the La Niña-like (cooler) Niño 3.4 index was associated with higher regional temperatures in summer, while the El Niño-like (warmer) Niño 3.4 index was associated with higher regional temperatures in fall and winter. A 1 K increase in Niño 3.4 index resulted in 0.74 °C (95%CI: -1.3 ~ -0.2 °C) decrease in temperature at 0-month lag during summer. During winter, a 1 K increase in the Niño 3.4 index led to a 1.50 ^o^C (95%CI: 0.7 ~ 2.3 °C) increase at a 7-month lag.

Region-stratified analyses suggest similar patterns in different reaches of the Yangtze River Basin. In the Upper reaches (Figure S3), Niño 3.4 index was positively associated with rainfall during spring (7–10 months lag), summer (3–4 months lag) and winter (9–12 months lag). Similarly, a significant positive association was observed during spring (0-month lag), summer (2–4 months lag), and winter (0–7 months lag) in the Middle and lower reaches (Figure S4). Moreover, the regional temperature was negatively associated with Niño 3.4 index during spring (0–1 months lag) and summer in the Middle and lower reaches (0–5 months lag) (Figure S5). The significant positive association between temperature and Niño 3.4 index was found during fall at 5–6 months lag in the Upper reaches (Figure S5) and during winter at 0–8 months lag in the Middle and lower reaches (Figure S6).

### Relationships between temperature, rainfall and bacillary dysentery

Both rainfall and temperature anomalies were associated with the burden of BD in the Yangtze River Basin at a monthly scale (Table 3). In comparison to the normal conditions, wetter than normal conditions were associated with 10.2% (IRR: 1.10, 95%CI: 1.06 ~ 1.13) increase in BD, and abnormally wet conditions were associated with 14.9% (IRR: 1.14, 95%CI: 1.10 ~ 1.18) increase in incidence of BD. Conversely, we found abnormally dry condition had protective effect on BD incidence (IRR: 0.91, 95%CI: 0.89 ~ 0.93). Additionally, warmer-than-normal and abnormally hot conditions were associated with 5.6% (IRR = 1.05, 95%CI: 1.03–1.08) and 6.2% (IRR: 1.06, 95%CI: 1.02 ~ 1.09) increase in incidence of BD respectively. While the abnormally cold conditions were associated with 11.2% (IRR: 0.88, 95%CI: 0.85 ~ 0.91) reduction in incidence of BD. Region-stratified results showed similar patterns in different reaches, but we found no significant association for abnormally hot conditions with BD in the Upper reaches.

**Table 3:** Incidence rate ratios (IRR) with 95% confidence intervals (CI) of bacillary dysentery associated with weather anomaly in Yangtze River Basin by different reaches

Weather anomaly	Cumulative *IRR* with 95%CI		
	Yangtze River basin	Upper reaches	Middle and lower reaches
Rainfall anomaly			
Normal	Ref	Ref	Ref
Abnormally dry	0.91 (0.89,0.93)	0.95 (0.91,0.99)	0.88 (0.86,0.90)
Dry	0.99 (0.97,1.01)	1.00 (0.96,1.03)	0.98 (0.96,1.01)
Wet	1.10 (1.06,1.13)	1.12 (1.08,1.16)	1.08 (1.03,1.14)
Abnormally wet	1.14 (1.10,1.18)	1.16 (1.11,1.20)	1.14 (1.09,1.20)
Temperature anomaly			
Normal	Ref	Ref	Ref
Abnormally cold	0.88 (0.85,0.91)	0.85 (0.80,0.90)	0.90 (0.86,0.94)
Cold	1.02 (0.99,1.04)	1.04 (0.99,1.09)	1.00 (0.97,1.03)
Warm	1.05 (1.03,1.08)	1.04 (1.01,1.07)	1.07 (1.02,1.11)
Abnormally hot	1.06 (1.02,1.09)	1.02 (0.98,1.06)	1.11 (1.06,1.16)

**Fig. 4:**
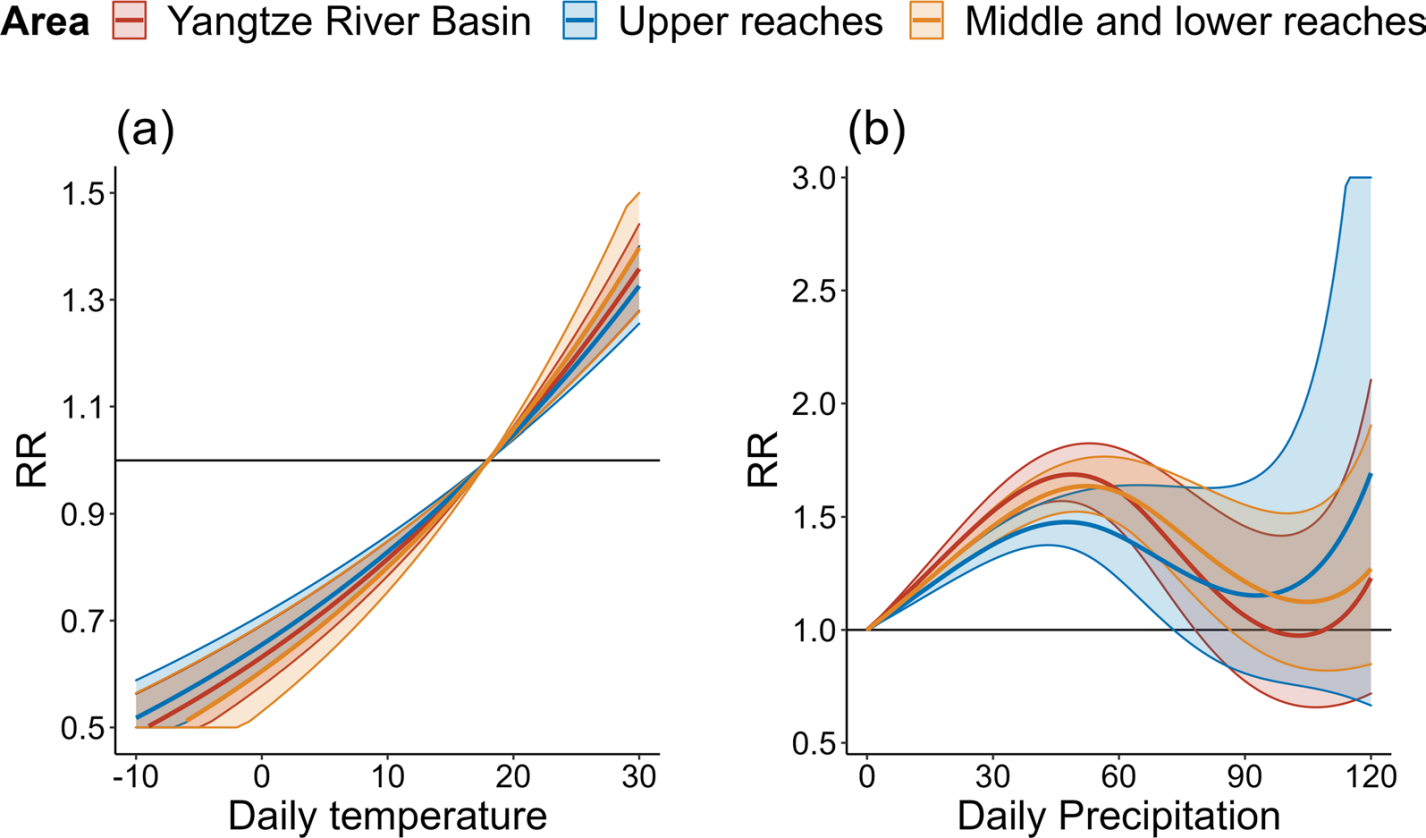
Pooled estimates of relative risks (RR) with 95% confidence interval (CI) of **a** daily max temperature (°C), **b** daily precipitation (mm) on bacillary dysentery for different reaches in Yangtze River basin. Reference: 50th percentile for temperature and 0 mm for precipitation

Pooled estimates of expose-response relationship between daily maximum temperature, daily total rainfall and BD in the Yangtze River Basin are shown in Fig. 4. We found that daily temperature was significantly associated with an increased risk of BD with an approximate linear trend. Furthermore, the pooled result of daily total rainfall on BD was an inverted U-shape curve. The risk of BD increased with increasing rainfall, but gradually decreased to non-significant when rainfall exceeded to a high level. The region-stratified analysis showed similar patterns of the association between BD and daily temperature, daily rainfall.

### Results of causal mediation analysis

**Fig. 5:**
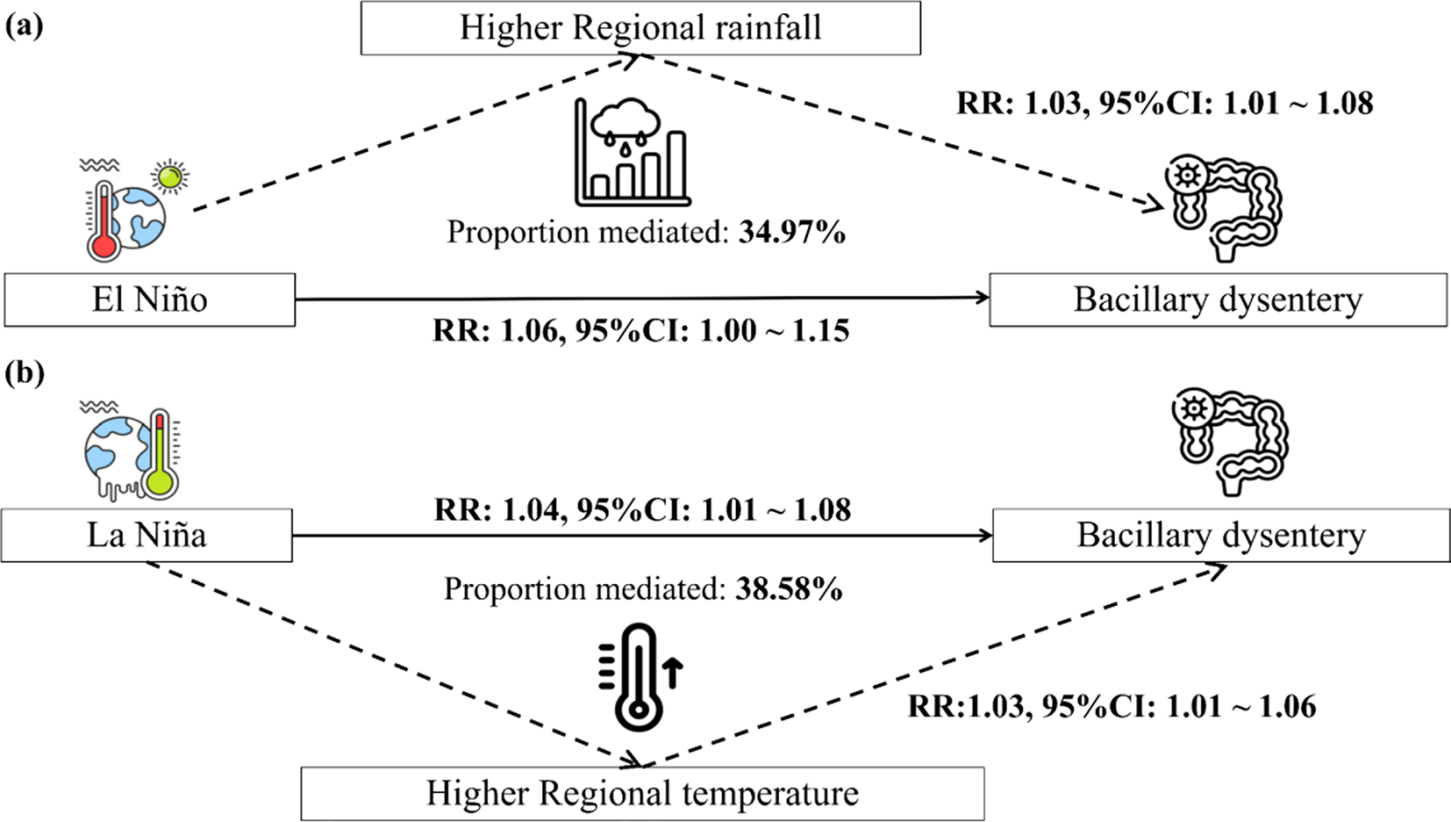
Schematic graphs of the mediation role and effect of different weather changes between the relationship of ENSO extreme events and bacillary dysentery in El-Niño (**a**) and La- Niña (**b**) conditions. Note: The solid arrows indicate the direct effect of ENSO event on bacillary dysentery, and the dashed arrows indicate the indirect effect of ENSO on bacillary dysentery that is attributable to rainfall and temperature. The models were mutually adjusted for each weather condition. Abbreviations: RR, rate ratios; CI, confidence interval

We further conducted the mediation analysis to examine the role of rainfall and temperature in the association between different ENSO events and the incidence of BD (Fig. 5, Table S3). In mediation analyses for the effect of El Niño events on BD considering rainfall, the estimated natural direct effect derived from the model was 1.06 (95%CI: 1.00 ~ 1.15), while the natural indirect effect was 1.03 (95%CI: 1.01 ~ 1.08). The mediation proportion was up to 34.97% (*p* < 0.001), meaning that increased regional rainfall following the warmer ENSO conditions served as a mediating factor in the associations between El Niño events and BD. Similarly, we found that heightened regional temperature following cooler ENSO conditions mediated in the associations between La Niña events and BD. The estimated natural direct effect was 1.04 (95%CI: 1.01 ~ 1.08) and the natural indirect effect was 1.03 (95%CI: 1.01 ~ 1.06), with the proportion of mediation was 38.58% (*p* < 0.05). We also ovserved a a negative mediation proportion when rainfall was mediated between La Niña and bacillary dysentery (− 12.63%), though the result was not statistically significant.

## Results of sensitivity analysis

In sensitivity analyses of time lags for ENSO events, we found there was a similar decresing trend between ENSO and BD. The strongest effect was observed within the current month (Table S2). The effect of El Niño event tends to last longer for significant association can be observed up to 2-month lag. While for the La Niña event, we found a significant decrease in the incidence of BD in the 3-month lag. Moreover, we performed sensitivity analyses by replacing the Niño 3.4 index with Niño 3 and Niño 4 index to examined the relationship between ENSO variability and local weather conditions. Results are shown in Supplementary Figs. S7 and S8, and these results using different ENSO index remained consistent to the results from using Niño 3.4 index. To justify the parameters we used in the models, we altered the df for the exposure space of temperature and rainfall, the df for the time trend. The pooled estimates of the temperature-BD, rainfall-BD association remained similar (Figure S9-S10). This suggests that the model was robust and insensitive to the parameters.

## Discussion

To the best of our knowledge, this is the first study to explore how ENSO influences the spread of bacillary dysentery in the Yangtze River Basin by affecting local weather patterns. The results of this study indicate that long-term climate variability ENSO was an important driver of BD, for a positive association with BD was found for both El Niño and La Niña events in the Yangtze River Basin. Moreover, we identified the potential exposure pathways for BD and the impact of weather conditions moderated by ENSO through mediation analyses, showing that BD risk was positively associated with increased regional rainfall caused by El Niño and increased regional temperature caused by La Niña.

Despite evidence in China remains limited, previous studies only focused on partial ENSO variability in a single region, with findings marked with uncertainty. In study conducted by Zhang et al. [20], SOI index was found negatively associated with BD in Shandong province. Studies conducted in Peru [51] and Nepal [17] also indicated that either El Niño or La Niña event can result in an increase in diarrhea. In contrast to these findings, our study demonstrated that both ENSO events can lead to an increase risk of BD. This may be due to the broad impact of ENSO variability on the climate conditions in the Yangtze River Basin, where both ENSO extremes were associated with anomaly weather [23]. Further regional stratified analysis showed that the impacts of ENSO variability on BD in the Middle and lower reaches of Yangtze River Basin was larger than that in the Upper reaches, where no significant association was found in latter for La Niña event. The divergence in the two reaches may reflect differences in climate-related exposure pathways from different ENSO events due to the weather sensitivity. Our findings provide empirical evidence of health deterioration from long-term climate variability, and underscore the importance of considering local weather teleconnections in exploring the impacts of ENSO.

Yangtze River Basin has been widely recognized as one of the ENSO tele-connected regions, and existing studies have found ENSO holds a significant impact on weather patterns such as temperature and precipitation anomalies [52]. Generally, we found variations in precipitation and temperature over the Yangtze River Basin were associated with SST anomalies but with different characteristics in different seasons. For example, we found SST anomalies was negatively associated with higher maximum temperature in spring and summer but positively associated in winter. The finding was in line with Tian et al. [53] which emphasized that in most of the river basin, temperature was negatively correlated with SST anomalies in summer. However, this relationship between ENSO and temperature is not stable for Xie et al. [54] noted that in summer, El Niño can lead to above-normal hot days in the southern part of the Yangtze River Basin. These findings suggested a complex underlying mechanism ENSO and regional temperature, which further study should focus on. Compared to temperature, the covariation of monthly rainfall with SST was more homogeneous in different seasons, for we found SST anomalies were positively associated with regional excessive rainfall in summer and winter, which was consistent with Jesse et al. [37] who found that Niño 3.4 index was positively correlated with monthly rainfall in southern China. Similar findings indicated that El Niño strongly intensified rainfall anomalies during June–August (JJA) and December-February (DJF) in the south and south-eastern part of China [55], and the flood disasters that occurred in the basin can be attributed to this [52]. With more frequent and intense weather anomaly moderated by ENSO have been observed under climate change [56], risk of climate-sensitive diseases within the Yangtze River Basin may further increase, therefore it’s important to implant more researches on evaluating the risk of infectious diseases due to weather changes brought by ENSO.

Our previous works have shown that both weather conditions and extreme weather event, such as temperature [57] and flood event [58] were closely associated with BD risk in cities located in the lower reaches of the Yangtze River Basin. In this study, based on the entire Yangtze River Basin, we provide further evidence that both increased regional temperature and rainfall were associated with a higher risk of BD on monthly and daily scale. On one hand, the exposure–response relationships we found between daily levels of weather and BD highlight the seasonal patterns and instant effect of such weather variables [40]. On the other hand, monthly weather anomalies can capture the shift of weather changes, including extreme weather events [59]. The different scales of weather conditions can better measure the patterns in the weather-BD relationships, emphasizing the important role of climate variability in heightening BD risk.

The mechanism behind the weather-BD association was well-documented in previous research [60]. We found similar patterns in the exposure–response relationship between temperature, precipitation, and BD incidence in both the Upper and Middle and lower reaches of the Yangtze River Basin, coinciding with studies in other regions [61]. The seasonal patterns revealed that the BD pathogen favors a warmer and wetter season probably because it’s more easily exposed to humans through faster replication, virulence gene activation, and longer survival time [39]. Moreover, rainfall surpluses can affect pathogens transport via the “run-off effect” by flushing fecal contaminated matter into groundwater, causing sewage overflow [18]. Several studies also pointed out floods can overwhelm water sanitation systems as well as cause forced displacement that leads to an outbreak of BD [62]. In contrast with a study in Taiwan [63], we found that abnormally cold and abnormally dry conditions can reduce the risk of BD, which may be because the environment is not suitable for the survival of Shigella in excessively cold or dry conditions.

On the basis of the association analyses, we identified possible weather pathways among ENSO events, weather conditions and BD through mediation analysis. During El Niño events, rainfall played a significant role in influencing the spread of Bacillary dysentery due to the increase and intensification of heavy rainfall events. In contrast, during La Niña events, temperature had a more notable impact on the disease for the rise of extreme temperature may promote the transmission of pathogen. We also found a negative a negative mediation proportion when rainfall was mediated between La Niña and bacillary dysentery. Previous research shows that ENSO's impact on BD is influenced by both regional climate and social vulnerability. Our study found La Niña conditions are linked to decreased rainfall and higher temperatures in the Yangtze River Basin, which could increase unsafe water consumption. However, La Niña’s mild rainfall may dilute pathogens, reducing BD risks. Conversely, El Niño's extreme rainfall events can promote BD transmission, highlighting the complexity of rainfall patterns and their effects on BD.

Studies elsewhere linked ENSO and infectious diseases based on the synchronous changes found in the ENSO-weather and weather-BD association [14], yet our findings provide an important advance by accounting for the specific weather changes moderated by ENSO events, which support the need of public health authority to adopt targeted measures to mitigate BD risk in the future. More importantly, compared to weather, large-scale climate variability like ENSO can contribute to building a more advanced early warning system for diarrhea in the Yangtze River Basin, considering the sub-seasonal to seasonal lead times it offers. The profound impacts of ENSO, compounded by climate change, underscore the necessity for comprehensive preparedness plans that integrate disease surveillance, disaster preparedness, and climate adaptation strategies [64]. It is crucial for public health authorities to assess potential ENSO-related health effects and the underlying mechanisms influenced by ENSO to implement effective and timely measures in response to the infectious disease risks associated with climate change [65].

Several limitations in our study should be acknowledged. First, the bacillary dysentery data we used in the study might be underreported because not all patients who are infected would seek medical services, thus may lead to an underestimate of the impact of ENSO events. Second, we chose two main weather variables instead of considering all meteorological factors in our study, though other weather variables have less effect on BD based on the previous evidence [66], potential exposure pathways between ENSO and BD might have been neglected. Third, we didn’t account for some confounders in our analyses, such as socioeconomic factors, personal behavior, and local immune level, which may lead to bias in our results.

## Conclusion

Our research provided further evidence that linked long-term climate variability and BD considering the weather pathways moderated by ENSO. We showed that both ENSO events can lead to an increased risk of BD in the Yangtze River Basin. Specifically, under El Niño event, warmer SSTs anomalies were associated with excessive rainfall and resulted in an increased risk of BD. While under La Niña event, cooler SSTs anomalies was associated with increased regional temperature that may drive the incidence of BD. These findings support the need for comprehensive awareness, advanced early warning systems, and targeted measures to respond to the risk of infectious diseases due to climate change.

## Supplementary Information

Additional file 1.

## Data Availability

The datasets generated and analysed during the study are not publicly available due to the policy of the Chinese Center for Disease Control and Prevention but are available from the corresponding author on reasonable request.

## Abbreviations

ENSO: El Niño-Southern Oscillation
BD: Bacillary dysentery
SST: Sea surface temperature
NOAA: National oceanic and atmospheric administration
IRR: Incidence rate ratio
CI: Confidence interval

## References

[1] Lee H, Calvin K, Dasgupta D, Krinner G, Mukherji A, Thorne P, et al. 2023: Climate Change 2023: synthesis Report, summary for policymakers. Contribution of Working Groups I, II and III to the Sixth Assessment Report of the Intergovernmental Panel on Climate Change. IPCC. 2023.

[2] Epstein PR. Climate change and human health. N Engl J Med. 2005;353(14):1433–6. 16207843 10.1056/NEJMp058079

[3] Hodges M, Belle JH, Carlton EJ, Liang S, Li H, Luo W, et al. Delays in reducing waterborne and water-related infectious diseases in China under climate change. Nat Clim Chang. 2014;4(12):1109–15. 25530812 10.1038/nclimate2428 PMC4266400

[4] Yi L, Xu X, Ge W, Xue H, Li J, Li D, et al. The impact of climate variability on infectious disease transmission in China: current knowledge and further directions. Environ Res. 2019;173:255–61. 30928856 10.1016/j.envres.2019.03.043

[5] Cai W, Santoso A, Wang G, Yeh S-W, An S-I, Cobb KM, et al. ENSO and greenhouse warming. Nat Clim Chang. 2015;5(9):849–59.

[6] McPhaden MJ, Zebiak SE, Glantz MH. ENSO as an integrating concept in earth science. Science. 2006;314(5806):1740–5. 17170296 10.1126/science.1132588

[7] Legler DM, Bryant KJ, O’brien JJ. Impact of ENSO-related climate anomalies on crop yields in the US. Clim Change. 1999;42(351):75.

[8] Liu Y, Cai W, Lin X, Li Z, Zhang Y. Nonlinear El Niño impacts on the global economy under climate change. Nat Commun. 2023;14(1):5887. 37735448 10.1038/s41467-023-41551-9 PMC10514271

[9] Hsiang SM, Meng KC, Cane MA. Civil conflicts are associated with the global climate. Nature. 2011;476(7361):438–41. 21866157 10.1038/nature10311

[10] Fisman DN, Tuite AR, Brown KA. Impact of El Niño Southern Oscillation on infectious disease hospitalization risk in the United States. Proc Natl Acad Sci. 2016;113(51):14589–94. 27791069 10.1073/pnas.1604980113 PMC5187703

[11] Semenza JC, Paz S. Climate change and infectious disease in Europe: impact, projection and adaptation. Lancet Reg Health-Europe. 2021;9:100238. 10.1016/j.lanepe.2021.100230 PMC8513157 34664039

[12] Wu X, Lu Y, Zhou S, Chen L, Xu B. Impact of climate change on human infectious diseases: empirical evidence and human adaptation. Environ Int. 2016;86:14–23. 26479830 10.1016/j.envint.2015.09.007

[13] Timmermann A, An S-I, Kug J-S, Jin F-F, Cai W, Capotondi A, et al. El Niño–southern oscillation complexity. Nature. 2018;559(7715):535–45. 30046070 10.1038/s41586-018-0252-6

[14] Xiao J, Liu T, Lin H, Zhu G, Zeng W, Li X, et al. Weather variables and the El Nino Southern Oscillation may drive the epidemics of dengue in Guangdong Province China. Sci Total Environ. 2018;624:926–34. 29275255 10.1016/j.scitotenv.2017.12.200

[15] Poveda G, Rojas W, Quiñones ML, Vélez ID, Mantilla RI, Ruiz D, et al. Coupling between annual and ENSO timescales in the malaria-climate association in Colombia. Environ Health Perspect. 2001;109(5):489–93. 11401760 10.1289/ehp.01109489 PMC1240308

[16] Pascual M, Rodó X, Ellner SP, Colwell R, Bouma MJ. Cholera dynamics and El Nino-southern oscillation. Science. 2000;289(5485):1766–9. 10976073 10.1126/science.289.5485.1766

[17] Adams N, Dhimal M, Mathews S, Iyer V, Murtugudde R, Liang X-Z, et al. El Niño Southern Oscillation, monsoon anomaly, and childhood diarrheal disease morbidity in Nepal. PNAS nexus. 2022;1(2):pgca032. 10.1093/pnasnexus/pgac032 PMC9802392 36713319

[18] Alexander KA, Heaney AK, Shaman J. Hydrometeorology and flood pulse dynamics drive diarrheal disease outbreaks and increase vulnerability to climate change in surface-water-dependent populations: a retrospective analysis. PLoS Med. 2018;15(11): e1002688. 30408029 10.1371/journal.pmed.1002688 PMC6224043

[19] Wu X, Liu J, Li C, Yin J. Impact of climate change on dysentery: scientific evidences, uncertainty, modeling and projections. Sci Total Environ. 2020;714: 136702. 31981871 10.1016/j.scitotenv.2020.136702

[20] Zhang Y, Bi P, Wang G, Hiller JE. El nino southern oscillation (Enso) and dysentery in Shandong Province China. Environ Res. 2007;103(1):117–20. 16690051 10.1016/j.envres.2006.03.005

[21] Zhang X, Gu X, Wang L, Zhou Y, Huang Z, Xu C, et al. Spatiotemporal variations in the incidence of bacillary dysentery and long-term effects associated with meteorological and socioeconomic factors in China from 2013 to 2017. Sci Total Environ. 2021;755: 142626. 33039932 10.1016/j.scitotenv.2020.142626

[22] Wolf J, Prüss-Ustün A, Cumming O, Bartram J, Bonjour S, Cairncross S, et al. Systematic review: assessing the impact of drinking water and sanitation on diarrhoeal disease in low-and middle-income settings: systematic review and meta-regression. Trop Med Int Health. 2014;19(8):928–42. 24811732 10.1111/tmi.12331

[23] Li X, Zhang K, Gu P, Feng H, Yin Y, Chen W, et al. Changes in precipitation extremes in the Yangtze river basin during 1960–2019 and the association with global warming, ENSO, and local effects. Sci Total Environ. 2021;760: 144244. 33348157 10.1016/j.scitotenv.2020.144244

[24] Yu J, Zhao L, Liang X-Z, Ho HC, Hashizume M, Huang C. The mediatory role of water quality on the association between extreme precipitation events and infectious diarrhea in the Yangtze River Basin China. Fundam Res. 2024;4(3):495–504. 38933184 10.1016/j.fmre.2023.05.019 PMC11197735

[25] Chen J, Wu X, Finlayson BL, Webber M, Wei T, Li M, et al. Variability and trend in the hydrology of the Yangtze river, China: annual precipitation and runoff. J Hydrol. 2014;513:403–12.

[26] Chen Y, Zhang S, Huang D, Li B-L, Liu J, Liu W, et al. The development of China’s Yangtze river economic belt: how to make it in a green way. Sci Bullet. 2017;62(9):648–51. 10.1016/j.scib.2017.04.009 36659306

[27] Wang J, Gao B, Stein A. The spatial statistic trinity: a generic framework for spatial sampling and inference. Environ Modell Softw. 2020;134: 104835.

[28] National Oceanic and Atmospheric Administration. Equatorial Pacific Sea surface temperatures. https://www.ncdc.noaa.gov/teleconnections/enso/indicators/sst. Accessed 15 November 2023.

[29] China Meteorological Administration. Identification method for El Nino/La Nina events. https://openstd.samr.gov.cn/bzgk/gb/newGbInfo?hcno=3E48085ABB8432F46774F96A1413CC38. Accessed 1 November 2023.

[30] Wu X, Fu Z, Deng G, Zhou J. Analysis on the operational status of direct network report system for infectious diseases. Appl Prev Med. 2013;1:26–8.

[31] National Health Commission. Diagnostic criteria for bacillary and amoebic dysentery. http://www.nhc.gov.cn/wjw/s9491/200802/39040/files/9c939b0b5de04a14be37e02421adc661.pdf. Accessed 12 November 2023.

[32] Oses N, Azpiroz I, Marchi S, Guidotti D, Quartulli M, Olaizola IG. Analysis of copernicus’ era5 climate reanalysis data as a replacement for weather station temperature measurements in machine learning models for olive phenology phase prediction. Sensors. 2020;20(21):6381. 33182272 10.3390/s20216381 PMC7664928

[33] Wang J-F, Zhang T-L, Fu B-J. A measure of spatial stratified heterogeneity. Ecol Ind. 2016;67:250–6.

[34] Wang J, Xu CD. Geodetector: principle and prospective. Acta Geogr Sin. 2017;72(1):116–34 (**(in Chinese)**).

[35] Wang JF, Li XH, Christakos G, Liao YL, Zhang T, Gu X, et al. Geographical detectors-based health risk assessment and its application in the neural tube defects study of the Heshun Region, China. Int J Geogr Inf Sci. 2010;24(1):107–27.

[36] Lee D, Chang HH, Sarnat SE, Levy K. Precipitation and salmonellosis incidence in Georgia, USA: interactions between extreme rainfall events and antecedent rainfall conditions. Environ Health Perspect. 2019;127(9): 097005. 31536392 10.1289/EHP4621 PMC6792369

[37] Anttila-Hughes JK, Jina AS, McCord GC. ENSO impacts child undernutrition in the global tropics. Nat Commun. 2021;12(1):5785. 34642319 10.1038/s41467-021-26048-7 PMC8511020

[38] Heaney AK, Shaman J, Alexander KA. El Niño-Southern oscillation and under-5 diarrhea in Botswana. Nat Commun. 2019;10(1):5798. 31862873 10.1038/s41467-019-13584-6 PMC6925142

[39] Liu Z, Tong MX, Xiang J, Dear K, Wang C, Ma W, et al. Daily temperature and bacillary dysentery: estimated effects, attributable risks, and future disease burden in 316 Chinese cities. Environ Health Perspect. 2020;128(5): 057008. 32452706 10.1289/EHP5779 PMC7266621

[40] Wang P, Goggins WB, Chan EY. A time-series study of the association of rainfall, relative humidity and ambient temperature with hospitalizations for rotavirus and norovirus infection among children in Hong Kong. Sci Total Environ. 2018;643:414–22. 29940452 10.1016/j.scitotenv.2018.06.189

[41] Antonio G. Modeling exposure–lag–response associations with distributed lag non-linear models. Stat Med. 2014;33(5):881–99. 24027094 10.1002/sim.5963 PMC4098103

[42] Morral-Puigmal C, Martínez-Solanas È, Villanueva CM, Basagaña X. Weather and gastrointestinal disease in Spain: a retrospective time series regression study. Environ Int. 2018;121:649–57. 30316180 10.1016/j.envint.2018.10.003

[43] Hayes AF, Preacher KJ. Statistical mediation analysis with a multicategorical independent variable. British J Mathem Stat Psychol. 2014;67(3):451–70. 10.1111/bmsp.12028 24188158

[44] Iacobucci D. Mediation analysis and categorical variables: the final frontier. J Consum Psychol. 2012;22(4):582–94. 10.1016/j.jcps.2012.03.009 PMC3501728 23180961

[45] Preacher KJ, Hayes AF. SPSS and SAS procedures for estimating indirect effects in simple mediation models. Behav res method instrum, comput. 2004;36:717–31. 10.3758/bf03206553 15641418

[46] Tingley D, Yamamoto T, Hirose K, Keele L, Imai K. Mediation: R package for causal mediation analysis. 2014.

[47] Bates M, Venables B, Team MRC. Package ‘splines’ R Version 2.0. 2011.

[48] Peng RD, Peng MRD. Package ‘tsModel’. Citeseer; 2010.

[49] Gasparrini A. Distributed Lag Linear and Non-Linear Models in R: the package dlnm. J Stat Softw. 2011;43(8):1–20. 22003319 PMC3191524

[50] Gasparrini A, Gasparrini MA. Package ‘mvmeta’. 2015. 10.1289/isee.2015.2015-3069

[51] Ramírez IJ, Grady SC. El Niño, climate, and cholera associations in Piura, Peru, 1991–2001: a wavelet analysis. EcoHealth. 2016;13:83–99. 26832694 10.1007/s10393-015-1095-3

[52] Chen Y, Zhao Y, Feng J, Wang F. ENSO cycle and climate anomaly in China. Chin J Oceanol. 2012;30(6):985–1000.

[53] Tian Q, Prange M, Merkel U. Precipitation and temperature changes in the major Chinese river basins during 1957–2013 and links to sea surface temperature. J Hydrol. 2016;536:208–21.

[54] Xie S-P, Du Y, Huang G, Zheng X-T, Tokinaga H, Hu K, et al. Decadal shift in El Niño influences on Indo–western Pacific and East Asian climate in the 1970s. J Clim. 2010;23(12):3352–68.

[55] An D, Eggeling J, Zhang L, He H, Sapkota A, Wang Y-C, et al. Extreme precipitation patterns in the Asia-Pacific region and its correlation with El Niño-southern oscillation (ENSO). Sci Rep. 2023;13(1):11068. 37422491 10.1038/s41598-023-38317-0 PMC10329631

[56] Huang P, Xie S. Mechanisms of change in ENSO-induced tropical Pacific rainfall variability in a warming climate. Nature Geosci. 2015;8(12):922–69.

[57] Hao Y, Liao W, Ma W, Zhang J, Zhang N, Zhong S, et al. Effects of ambient temperature on bacillary dysentery: a multi-city analysis in Anhui Province China. Sci Total Environ. 2019;671:1206–13. 31186130 10.1016/j.scitotenv.2019.03.443

[58] Zhang N, Song D, Zhang J, Liao W, Miao K, Zhong S, et al. The impact of the 2016 flood event in Anhui Province, China on infectious diarrhea disease: an interrupted time-series study. Environ Int. 2019;127:801–9. 31051323 10.1016/j.envint.2019.03.063

[59] Dimitrova A, McElroy S, Levy M, Gershunov A, Benmarhnia T. Precipitation variability and risk of infectious disease in children under 5 years for 32 countries: a global analysis using demographic and Health Survey data. Lancet Planet Health. 2022;6(2):e147–55. 35150623 10.1016/S2542-5196(21)00325-9

[60] Levy K, Woster AP, Goldstein RS, Carlton EJ. Untangling the impacts of climate change on waterborne diseases: a systematic review of relationships between diarrheal diseases and temperature, rainfall, flooding, and drought. Environ Sci Technol. 2016;50(10):4905–22. 27058059 10.1021/acs.est.5b06186 PMC5468171

[61] Xiao G, Xu C, Wang J, Yang D, Wang L. Spatial-temporal pattern and risk factor analysis of bacillary dysentery in the Beijing-Tianjin-Tangshan urban region of China. BMC Public Health. 2014;14:998. 25257255 10.1186/1471-2458-14-998 PMC4192281

[62] Levy K, Smith SM, Carlton EJ. Climate change impacts on waterborne diseases: moving toward designing interventions. Current Environ Health Rep. 2018;5:272–82. 10.1007/s40572-018-0199-7 PMC6119235 29721700

[63] Andhikaputra G, Sharma A, Sapkota A, He H, Lin Y-K, Deng L-W, et al. Quantifying the effects of anomalies of temperature, precipitation, and surface water storage on diarrhea risk in Taiwan. Epidemiol Health Sec. 2023;45:e2023024. 10.4178/epih.e2023024 PMC10396799 36822193

[64] Ortiz-Prado E, Camacho-Vasconez A, Izquierdo-Condoy JS, Bambaren C, Hernández-Galindo L, Sanchez JC. El Niño-southern oscillation: a call to action for public health emergency preparedness and response. The Lancet Reg Health-Am. 2023;27:100601. 37766923 10.1016/j.lana.2023.100601 PMC10520419

[65] Haines A, Lam HC. El Niño and health in an era of unprecedented climate change. The Lancet. 2023;402(10415):1811–3. 10.1016/S0140-6736(23)01664-1 37597524

[66] Zhang H, Si Y, Wang X, Gong P. Patterns of bacillary dysentery in China, 2005–2010. Int J Environ Res Public Health. 2016;13(2):164. 26828503 10.3390/ijerph13020164 PMC4772184

