# Supplementary material for: El Niño southern oscillation, weather patterns, and bacillary dysentery in the Yangtze River Basin, China
Source: Glob Health Res Policy. 2024 Nov 11;9:45. doi: 10.1186/s41256-024-00389-4 (PMC11552299; doi:10.1186/s41256-024-00389-4)
Supplement: Supplementary file 1 — Additional file 1. [file 41256_2024_389_MOESM1_ESM.docx]

**Additional file 1**

**El Niño Southern Oscillation, weather patterns, and bacillary dysentery in the Yangtze River Basin, China**

Caiji Li ^1#^, Xiaowen Wang^2 #^, Zehua Liu^3^, Liangliang Cheng^3^, Cunrui Huang ^1, 3 *^, Jing Wang^3**^

**Author affiliations**

^1^ School of Public Health, Sun Yat-sen University, Guangzhou 510080, China;

^2^ Department of Nutrition, Harvard TH Chan School of Public Health, Boston, MA 02115, USA.

^3^ Vanke School of Public Health, Tsinghua University, Beijing 100084, China;

*** Corresponding author:**. Prof. Cunrui Huang, Vanke School of Public Health, Tsinghua University, Haidian District, Beijing 100084, China

**^**^ Corresponding author:** Dr. Jing Wang, Vanke School of Public Health, Tsinghua University, Haidian District, Beijing 100084, China.

**Email address:** huangcunrui@mail.tsinghua.edu.cn (Cunrui Huang), jingjing0330@mail.tsinghua.edu.cn (Jing Wang).

^#^These authors contributed equally to this work.

**Table S1.** The *q*-statistic values of meteorological factors in upper, middle and lower reaches of the Yangtze River Basin.

| **Meteorological factors** | **Upper reaches** | **Middle and lower reaches** |
| --- | --- | --- |
| Total rainfall | 0.19* | 0.17* |
| Maximum temperature | 0.11* | 0.20* |
| Relative humidity | 0.04 | 0.06 |

Note: * Denotes statistically significant.

**Table S2.** Comparison of incidence rate ratios (IRR) and 95% confidence intervals (CI) of cumulative 0-3 month lags in the effect of ENSO events on BD incidence in different reaches from Yangtze River Basin.

| **ENSO** | **Region** | **Cumulative *IRR* (95%CI)** | | | |
| --- | --- | --- | --- | --- | --- |
|  |  | Lag 0 | Lag 0-1 | Lag 0-2 | Lag 0-3 |
| Neutral | Reference | - | - | - | - |
| El Niño | Yangtze River Basin | **1.06 (1.04,1.08)** | **1.04 (1.02,1.06)** | **1.03 (1.01,1.05)** | 1.01 (0.98,1.02) |
|  | Upper reaches | **1.03 (1.00,1.06)** | 1.01 (0.98,1.04) | 1.01 (0.98,1.04) | 0.99 (0.96,1.02) |
|  | Middle and lower reaches | **1.08 (1.06,1.11)** | **1.06 (1.03,1.09)** | **1.04 (1.01,1.07)** | **1.02 (1.01,1.04)** |
| La Niña | Yangtze River Basin | **1.03 (1.02,1.05)** | 1.01 (0.97,1.03) | 0.99 (0.97,1.01) | **0.97 (0.95,0.99)** |
|  | Upper reaches | 1.01 (0.99,1.03) | 0.99 (0.97,1.02) | 0.98 (0.95,1.01) | **0.96 (0.93,0.99)** |
|  | Middle and lower reaches | **1.05 (1.03,1.07)** | **1.02 (1.00,1.05)** | 1.01 (0.98,1.03) | 0.98 (0.96,1.00) |

**Table S3.** Mediation effects of temperature and rainfall on the association between ENSO events and BD.

| **Mediators** | **El Niño** | **La Niña** |
| --- | --- | --- |
| **Monthly total rainfall** |  |  |
| Total effect | 1.11 (1.04,1.17)* | 1.03 (1.01,1.05)* |
| Natural direct effect | 1.06 (1.00,1.15) ^a^ | 1.04 (1.02,1.06)* |
| Natural indirect effect | 1.03(1.01,1.08)* | 0.95 (0.84,1.06) |
| Proportion mediated,% | 34.97%* | -12.63% |
| **Monthly maximum temperature** |  |  |
| Total effect | 1.10 (1.01,1.20) ^a^ | 1.07 (1.02,1.13)* |
| Natural direct effect | 1.10 (1.01,1.19) ^a^ | 1.04 (1.01,1.08) ^a^ |
| Natural indirect effect | 0.99 (0.98,1.01) | 1.03 (1.01,1.06) ^a^ |
| Proportion mediated,% | -0.23% | 38.58%^a^ |

**Note:** ^a^: *P* < 0.05; ^*^: *P* < 0.001. The models are constructed separately for El Niño and La Niña event.


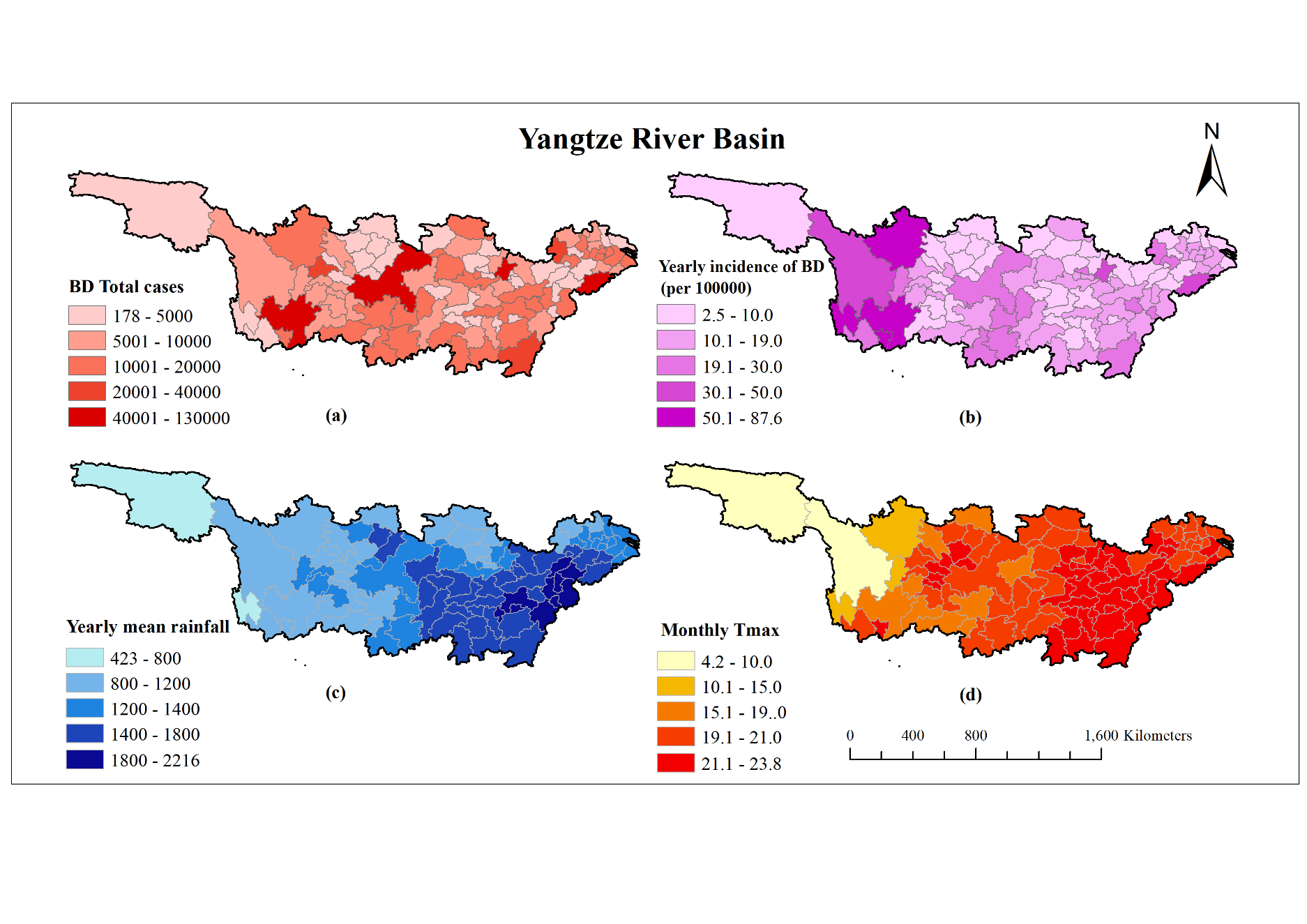


**Figure S1.** The spatial distribution of the cumulative cases (a) and yearly mean incidence (b) of bacillary dysentery, yearly mean rainfall (c), and the yearly monthly maximum temperature (d) in the Yangtze River Basin from 2005-2020.


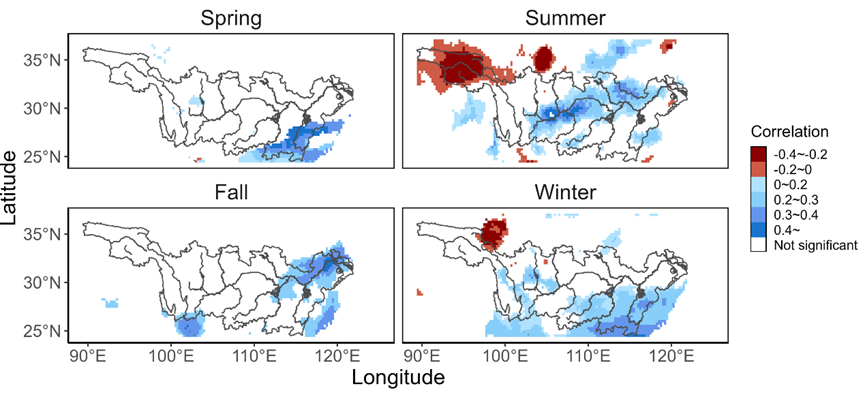


**Figure S2.** Correlation map between Niño 3.4 index and grid-level monthly total rainfall in Yangtze River basin stratified by different seasons from 2005-2020.


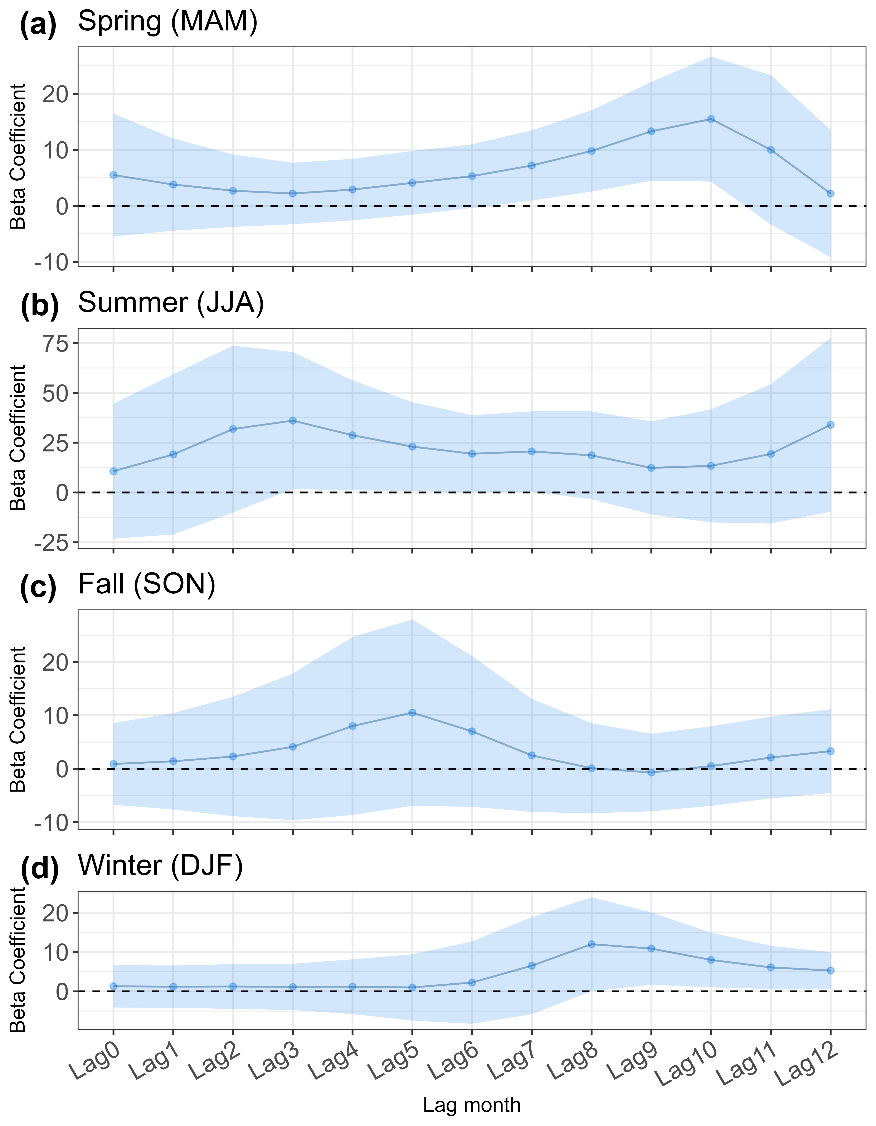


**Figure S3.** Associations between Niño 3.4 anomalies and total regional rainfall (mm) in the Upper reaches of Yangtze River basin by different season from 2005 to 2020.


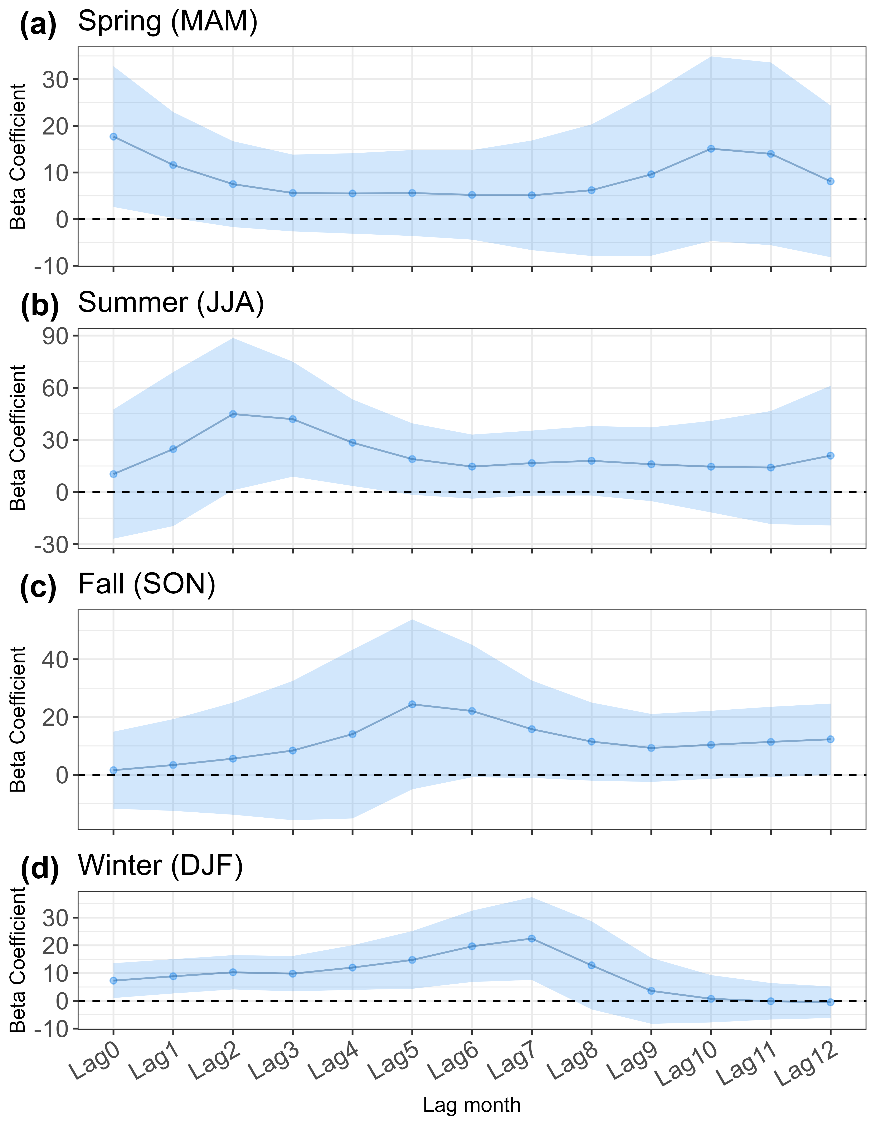


**Figure S4.** Associations between Niño 3.4 anomalies and total regional rainfall (mm) in the Middle and lower reaches of Yangtze River basin by different season from 2005 to 2020.


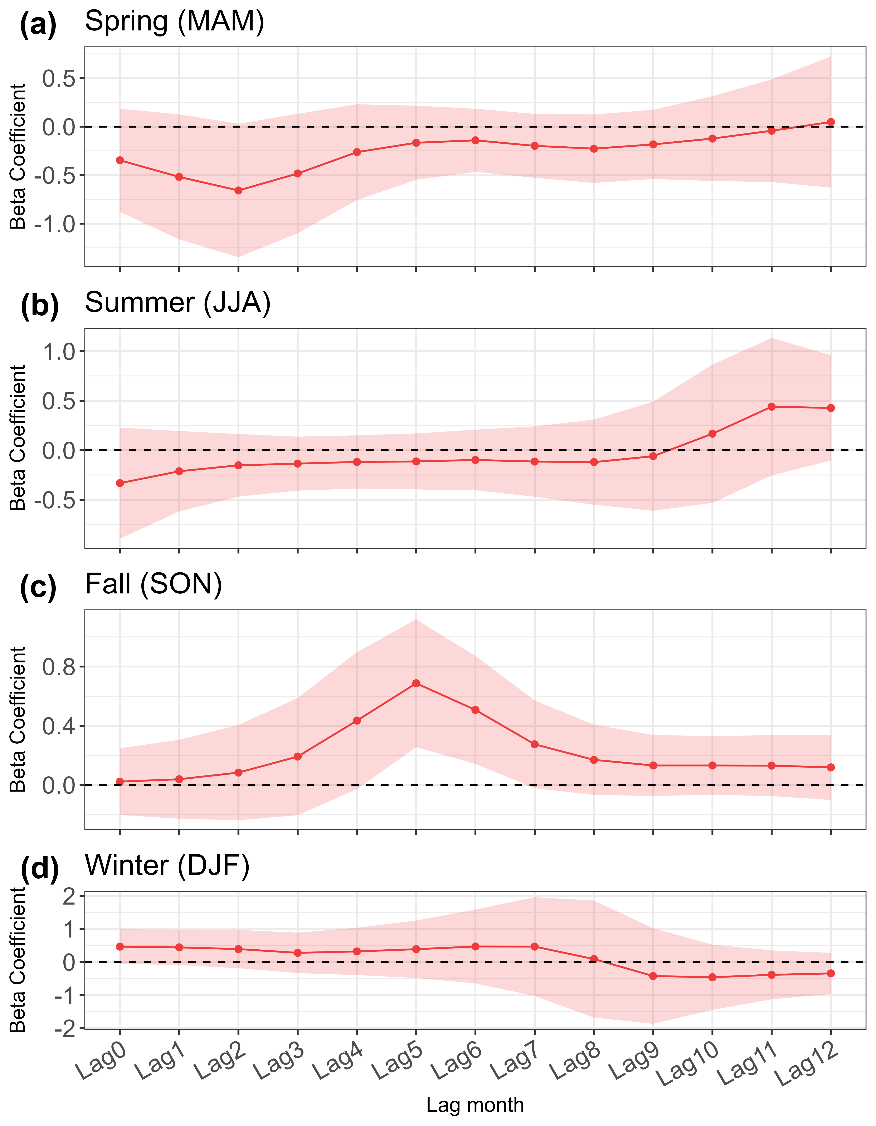


**Figure S5.** Associations between Niño 3.4 anomalies and monthly maximum temperature (℃) in the Upper reaches of Yangtze River basin by different season from 2005 to 2020.


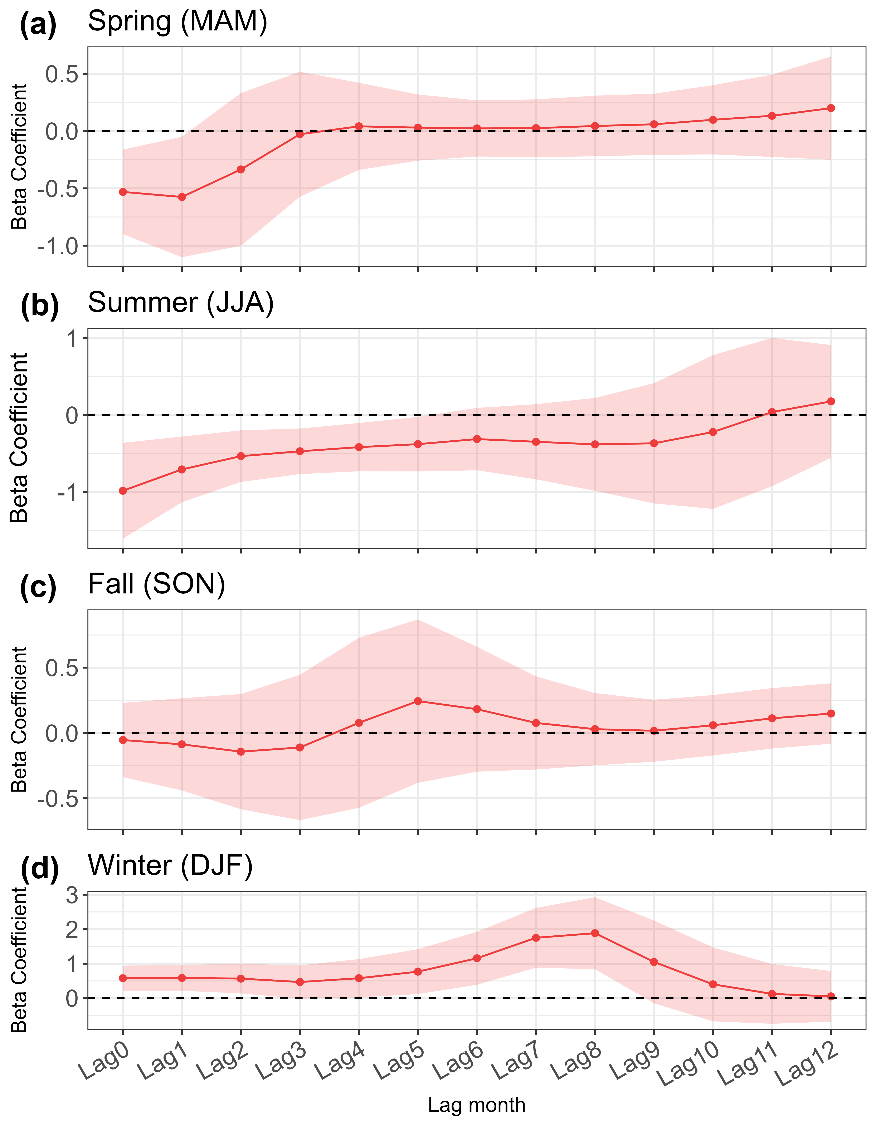


**Figure S6.** Associations between Niño 3.4 anomalies and monthly maximum temperature (℃) in the Middle and lower reaches of Yangtze River basin by different season from 2005 to 2020.


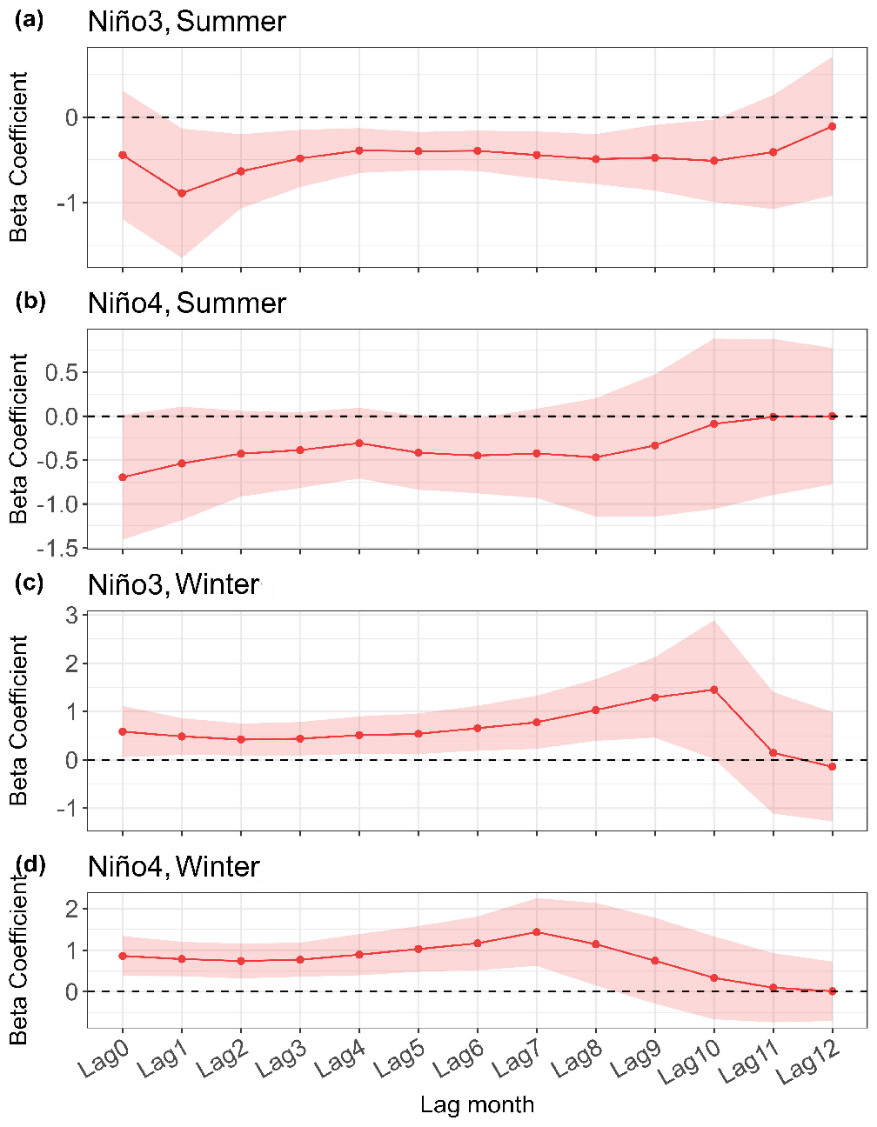


**Figure S7.** Summer and winter monthly average maximum temperature (℃) associated with Niño 3 index, Niño 4 index from 2005 to 2020 in the Yangtze River basin.


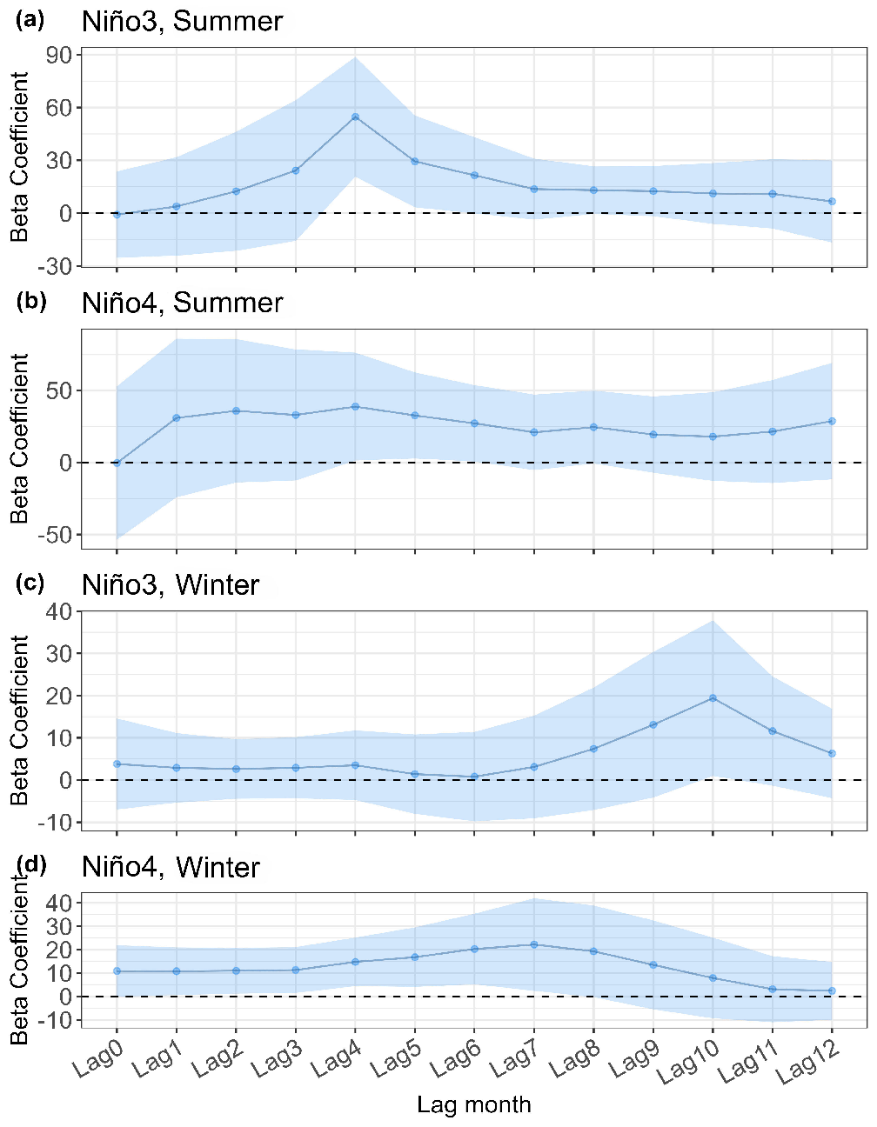


**Figure S8.** Summer and winter monthly total rainfall (mm) associated with Niño 3 index, Niño 4 index from 2005 to 2020 in the Yangtze River basin.


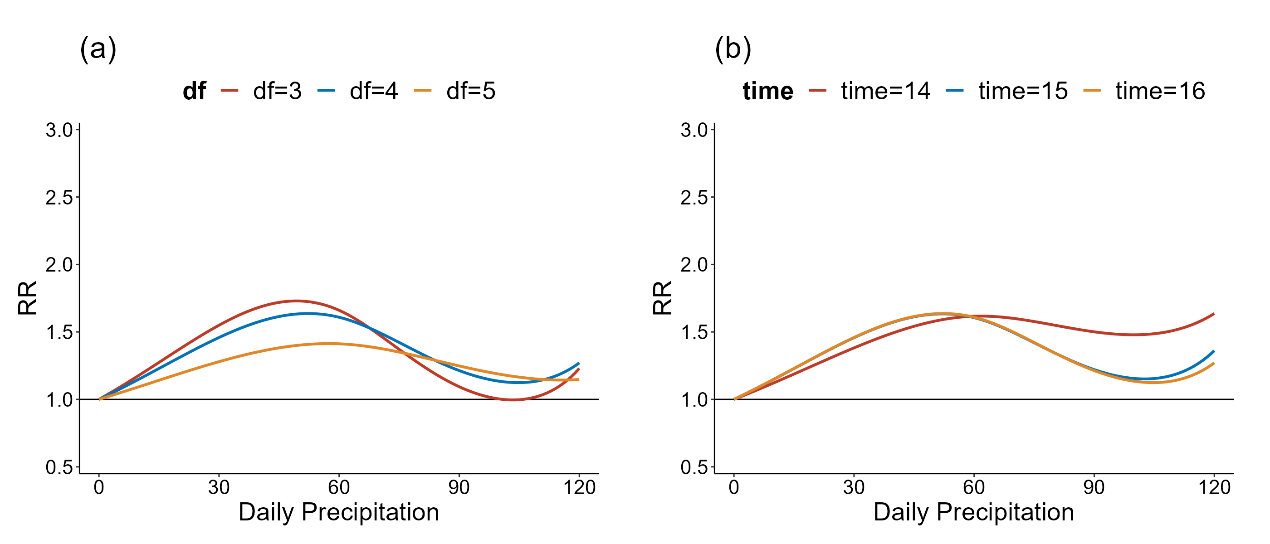


**Figure S9.** Pooled estimates of rainfall on bacillary dysentery when changing the *df* of rainfall (a) and time trend (b) for the exposure space of the cross-basis function.


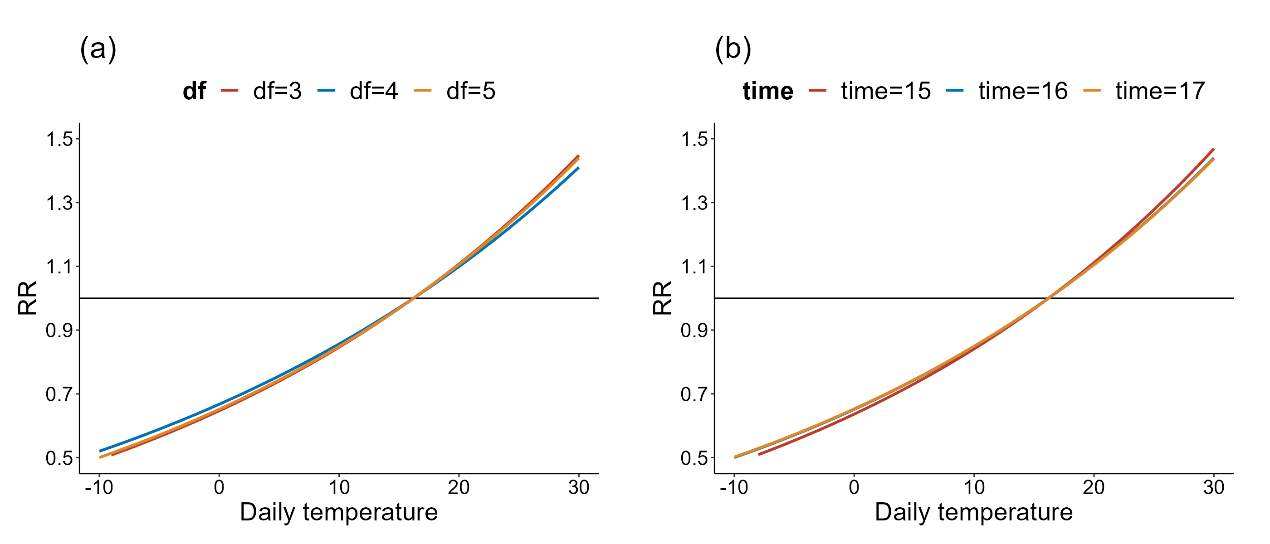


**Figure S10.** Pooled estimates of daily maximum temperature on bacillary dysentery when changing the *df* of Tmax (a) and time trend (b) for the exposure space of the cross-basis function.
